# Determination of parasitic burden in the brain tissue of infected mice in acute toxoplasmosis after treatment by fluconazole combined with sulfadiazine and pyrimethamine

**DOI:** 10.1186/s40001-021-00537-3

**Published:** 2021-06-30

**Authors:** Sekandarpour Sina, Jafari Modrek Mohammad, Shafiei Reza, Mohammadiha Anita, Etemadi Soudabeh, Mirahmadi Hadi

**Affiliations:** 1 Infectious Disease and Tropical Medicine Research Center, Resistance Tuberculosis Institute, Zahedan University of Medical Sciences, Zahedan, Iran; 2 Department of Medical Parasitology and Mycology, Faculty of Medicine, School of Medicine, Zahedan University of Medical Sciences, Zahedan, Iran; 3 Vector-Borne Diseases Research Center, North Khorasan University of Medical Sciences, Bojnurd, Iran; 4 Department of Parasitology, Faculty of Medical Sciences, Tarbiat Modares University, Tehran, Iran

**Keywords:** Real-time PCR, Parasitic burdens, Drug, *Toxoplasma gondii*

## Abstract

**Background/aims:**

One of the opportunistic pathogens which cause serious problems in the human immune system is *Toxoplasma gondii,* with toxoplasma encephalitis (TE) seen in patients affected by it. The treatment of these patients is limited, and if not treated on time, death will be possible.

**Methods:**

In this study, the effects of the treatment with different doses of fluconazole (FLZ) in combination with the current treatment of acute toxoplasmosis on reducing the mortality rate and the parasitic load in the murine model in vivo were studied. The mice were treated with different doses of fluconazole alone, sulfadiazine, and pyrimethamine plus fluconazole. A day after the end of the treatment and 1 day before death, the mice’s brains were collected, and after DNA extraction and molecular tests, the parasite burden was detected.

**Results:**

This study showed that a 10-day treatment with 20 mg/kg of fluconazole combined with sulfadiazine and pyrimethamine 1.40 mg/kg per day affected acute toxoplasmosis and reduced the parasitic load significantly in brain tissues and also increased the survival rate of all mice in this group until the last day of the study, in contrast to other treatment groups. These results also indicate the positive effects of combined therapy on *Toxoplasma gondii* and the prevention of relapse.

**Conclusions:**

Reducing the parasitic burden and increasing the survival rate were more effective against acute toxoplasmosis in the combined treatment of different doses of fluconazole with current treatments than current treatments without fluconazole. In other words, combination therapy with fluconazole plus pyrimethamine reduced the parasitic burden in the brain significantly, so it could be a replacement therapy in patients with intolerance sulfadiazine.

## Introduction

*Toxoplasma gondii* (*T. gondii*) is an obligate intracellular parasite with a global spread that one-third of the world’s human population harboring toxoplasmosis. Warm-blooded animals and humans are the hosts of this parasite [1]. This parasite is transmitted to new hosts by consuming undercooked meat contaminated with tissue cysts, oocysts excreted in cats’ feces, or the congenital transmission of the active form of the parasite [2]. Like several protozoa infections, toxoplasmosis in people with an efficient and self-limiting immune system is usually mild, but in patients with a deficient immune system and a fetus, it may be life-threatening [3, 4]. In addition, in patients with a deficient immune system, it leads to severe clinical signs, such as *Toxoplasma* encephalitis (TE), which in severe cases can be fatal [5]. In this case, the most effective treatment is the combination of sulfadiazine (SDZ) and pyrimethamine (PYR) which disrupts the synthesis of folic acid and reduces its level in tachyzoites [6]. This treatment is highly effective but causes side effects, such as hematological disorders and extreme sensitivity, particularly in patients with immune deficiencies. These types of drugs are not effective against cysts present in the tissues and lead to the re-emergence of the disease [7]. Considering the limited anti *toxoplasma* agents, many attempts have been made to discover novel agents with high efficacy and safety profiles [8].

In 1990, azole compounds entered the market for the treatment of systemic fungal infections, with minimal side effects [9]. For example, *Cryptococcus neoformans* is an opportunistic pathogen of the CNS and is the most common cause of meningitis in HIV patients*.* According to the previous document, mixed infections have been observed in *T. gondii, Candida spp, or Cryptococcosis* in HIV patients [10].

Fluconazole (FLZ) plays a crucial role in treating *Candida*, being the appropriate medication for eliminating the infection [11]. One of the suitable and practical methods for detecting the parasite burden and the quantity of parasites is the use of the Real-time Quantitative PCR (QPCR) that evaluates the quantity of *T. gondii* in mice tissues [12, 13]. In the present study, the effects of the SDZ/PYR combination were examined together with FLZ as well as FLZ with PYR on the parasite burden in the brain tissues of mice by the real-time q PCR method.

## Materials and methods

### Parasites

The RH strain of *T. gondii* was utilized in this investigation. After 72–96 h of intraperitoneal (i. p.) injection with 0.5 ml of the parasite suspension in sterile phosphate-buffered saline (PBS; pH = 7.4), fresh tachyzoites were harvested from the peritoneal cavity of Swiss-Webster mice. The tachyzoites were washed twice in PBS containing 100 IU/ml penicillin and 100lg/ml streptomycin, and then, the peritoneal cells and debris were removed by centrifuging them at 200×*g* for 10 min at 4 °C. The number of parasites was determined (8–12 × 107/ml) by counting them in a hemacytometer under light microscopy (× 400) [14].

### Mice

Inbred female BALB/c mice, weighing 18–25 g, were exposed to environmental conditions to adapt to them (temperature 2 ± 23 °C, 12 h light: 12-h darkness, humidity 40%) for a week. When adapted, the mice were divided randomly into nine subgroups (*n* = 10 per cage). The project underwent an ethical review and was approved by the Ethics Committee of Zahedan University of Medical Sciences.

### Drug

PYR and FLZ were purchased from Santa Cruz (Santa Cruz Biotechnology), and SDZ and polyethylene glycol were purchased from Sigma (Sigma-Aldrich). For in vivo tests, the drugs were diluted in polyethylene glycol (PEG) mol wt 200 (Sigma-Aldrich).

### Experimental design

The treatment groups contained different doses of FLZ alone (FLZ 10 and 20 mg/kg/day) in 2 groups, one group with SDZ and PYR alone, 2 groups with SDZ and PYR combined with FLZ (FLZ10 and 20 mg/kg/day + SDZ/PYR 40/1 mg/kg/day), 3 groups with PYR plus FLZ (FLZ 20 and 40, 80 + PYR 10 mg/kg/day), and only the untreated control group were divided [15]. The mice were infected with $1 \times 10^{4}$ tachyzoites intraperitoneally. Treatment was given in different experimental groups 24 h after inoculation for 10 days by oral gavage. One day after the end of the therapy, five mice from each group were killed, and the remaining ones were monitored for 30 days. After separation and cleaning, the mice tissues were stored at − 70 °C.

### DNA extraction of tissues

All mice’s brains tachyzoites were collected. Next, the DNA of tissues and tachyzoites was extracted using the Dyna Bio Blood/Tissue DNA Extraction Mini Kit (Cat. No.: L-94044).

**Fig. 1:**
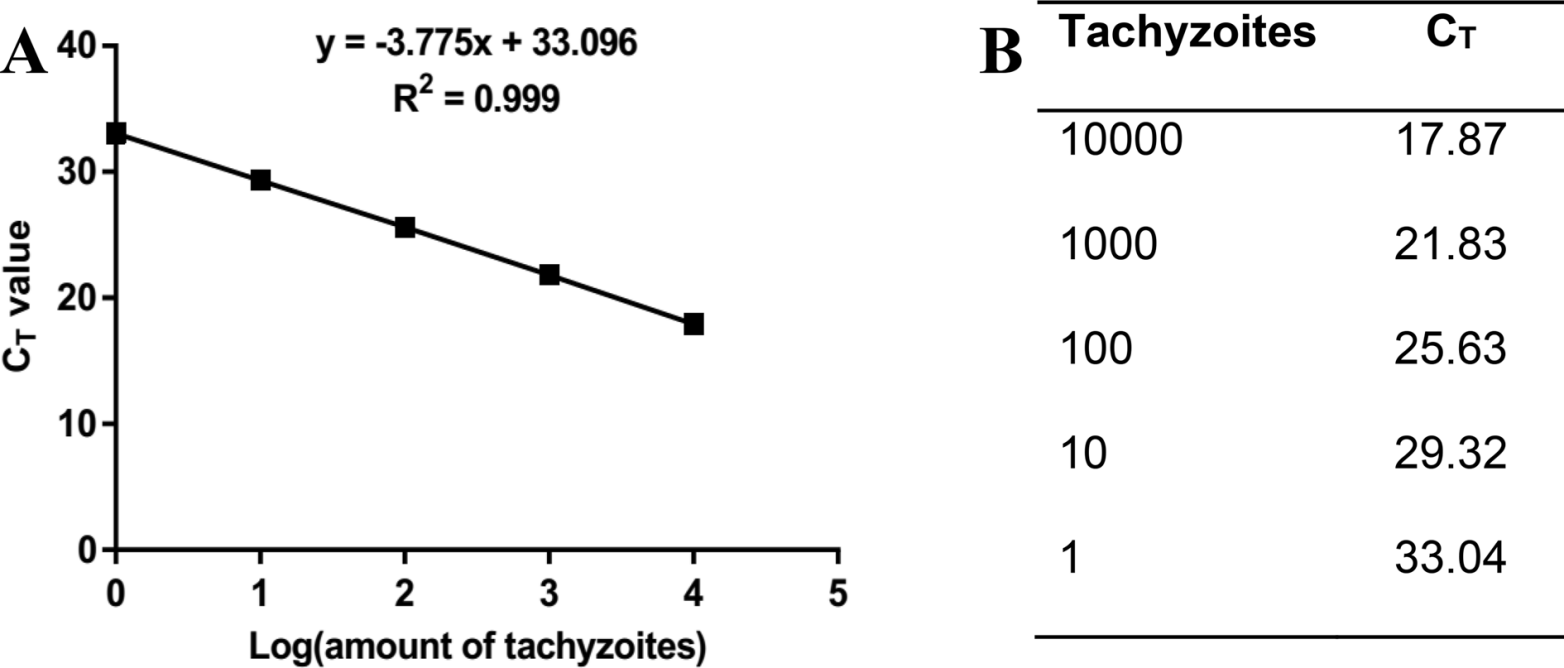
Establishment of the standard curve for quantification of *T. gondii*; serial dilutions of *T. gondii* DNA, ranging from 10,000 to 1 tachyzoite, was used as the template for real-time PCR analysis. **A** *C*_T_ values were plotted against tachyzoites’ value. **B** *C*_T_ values for all data points

The target DNA for the real-time PCR amplification was the published sequence of the 35-fold repetitive B1 gene of the *T. gondii* RH strain (Gen Bank: AF179871.1). Next, 5 µl of the template DNA was added to a reaction mixture containing 12.5 µl 2 × q PCR Master Mix Green-High Rox (Ampliqon. Denmark), 2 µl forward primer TOXO F (2 µl, 5′-TCCCCTCTGCTGGCGAAAAGT-3′), 2 µl reverse primer TOXO R (2 µl, 5′-AGCGTTCGTGGTCAACTATCGATTG-3′) and 3.5 µl DEPC-Distilled water in a final volume of 25 µl. The qPCR was performed via the StepOne™ Real-Time PCR System (applied bio-systems). The reaction was carried out in 10 min at 95 °C, 40 cycles at 95 °C for 20 s (denaturation), 58.5 °C for 30 s (annealing), 72 °C for 30 s (amplification), followed by a final extension for 5 min at 72 °C. Based on the SYBR green assay, the annealing temperature of the primer pair was optimized by temperature gradient [16]. The melting curve analysis verified the correct product size, and it did not result in the formation of side products or primer dimmers. The samples were run in duplicate in two independent experiments, and the means were calculated. The number of parasites in the samples was calculated using the QPCR cycle threshold (*C*_T_) value according to a standard curve (the linear curve slope: − 3.7, *Y* intercept: 33.096, *R*^2^: 0.99), which was obtained from DNA samples of a range of serial dilutions tenfold ($1 \times 10^{4} - 1$/ml) of RH strain tachyzoites (Fig. 1). The results were expressed as *T. gondii* tachyzoite equivalents per mg of the tissue.

### Survival analysis

For survival studies, the mice were injected intraperitoneally (i.p.) with 10^4^ tachyzoites of the RH strain of *T. gondii*. The treatment was started 1 day after the infection, and 10 mice were kept in one cage in each treatment group. The treatment continued for 10 days, and the mice were administered orally once a day. The untreated control group received the PEG solvent. Within 40 days, the mortality rate of the studied groups was monitored, and after collecting the survival curve information, the Kaplan–Meier estimator was utilized, with the survival curves compared using the log-rank (Mantel–Cox) test in Graphpad Prism 6.0 (GraphPad Software Inc.). *P* ≤ 0.05 was considered statistically significant.

### Statistical analysis

The collected data were statistically analyzed by SPSS 18 software. The Kruskal–Wallis test was used to compare DNA quantities among tissues and days of observation at the significance level of 0.05. The Mann–Whitney *U* test was also utilized for the multiple comparisons of the means between every two tissues or every 2 days with the Bonferroni correction changing the significance level to 0.01 and 0.025.

## Results

**Fig. 2:**
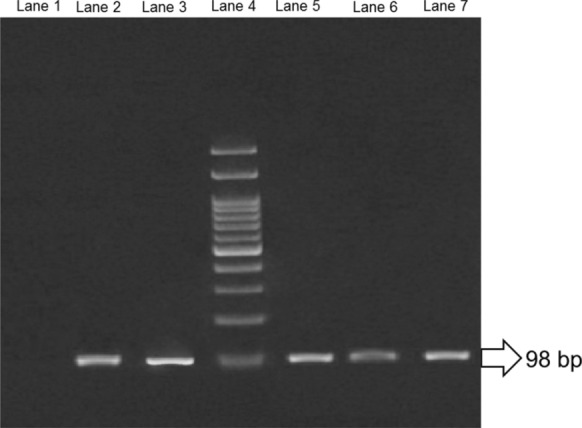
QPCR amplification products analyzed by agarose gel electrophoresis (2%). Lane 3 positive control (standard DNA was extracted from RH strain tachyzoites), Lane 2, 5, 6, 7: QPCR products of unknown samples from tissues, Lane 1: negative control, Lane 4: DNA molecular weight marker (100 bp)

The QPCR analysis of data which included the 1 day after the end of medication administration and the day before death could determine the exact number of *T. gondii* in tissues of all infected animals in the control group as untreated and groups of undertaken therapy. Using QPCR and amplification fragment 98 bp, the B1 gene of *T. gondii* was successful (Fig. 2).

### Effect of different doses of FLZ

The treatment with doses 10 and 20 mg/kg/day of FLZ of mice infected with $1 \times 10^{4}$ tachyzoites of the RH strain and the difference in the survival of this group was compared with the control group; all mice died before the 10th day. However, a significant difference was observed, after comparing the copy number of the DNA parasites of these groups with that of the non-treated group (Table 1).

**Table 1:** The parasite burden in different groups of treatment by various doses of FLZ

Groups	Last day (before death)
Untreated	11,084,800
FLZ 10 mg	1,629,200
*P* value	*P* < 0.009
Untreated	11,084,800
FLZ 20 mg	1,520,400
*P* value	*P* < 0.009

The results of the mean and standard deviation of the quantity of DNA were obtained from three different treatment groups. The Kruskal–Wallis test was used for the comparison between the groups at the significance level of 0.05. The Mann–Whitney *U* test was used for multiple comparisons between the groups with the Bonferroni correction changing the significance level to 0.01 and 0.025. The parasite load of *Toxoplasma* tachyzoites in each of the two groups indicated a statistically significant difference

**Fig. 3:**
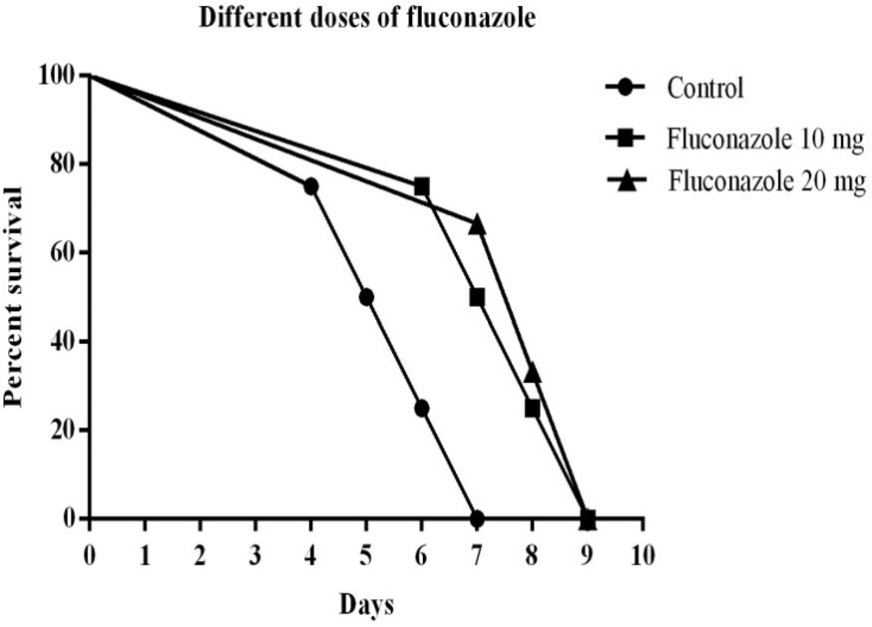
Effect of a 10-day treatment with different doses of FLZ on the survival of female BALB/c mice acutely infected with 10,000 tachyzoites of *T. gondii* RH strain. The results were evaluated by Kaplan–Meier product-limit method. *P* < 0.001 for the different doses of FLZ. Control; (*n* = 5); FLZ 10 (*n* = 5); FLZ 20 (*n* = 5)

Survival results also indicated that the treatment of different doses of FLZ alone was not capable of increasing the longevity of the treated mice compared with the untreated ones, and the mice died before the end of the 10-day treatment period (Fig. 3).

### Combination of FLZ plus SDZ/PYR

The combination of different doses of FLZ, in addition to SDZ/PYR, resulted in the significant prevention of the death of the treated group compared to the non-treated one. In addition, a significant decrease was observed in the level of the parasite burden in the treated group compared to the untreated one. All mice in this group survived 40 days after the intraperitoneal inoculation of parasites, and the lowest parasitic load was found in this group at the presence of the combination of FLZ 20 mg/kg/day plus SDZ/PYR 40/1 mg/kg/day.

In groups with the doses of 10 mg of FLZ in combination with SDZ/PYR as well as the treatment combined with SDZ and PYR without FLZ, a reduction was detected in the number of the parasites in brain tissues. The highest DNA copy number was found in the group treated with SDZ/PYR alone among the groups with different doses of FLZ. The effects of the medication in different days of sampling the days before death showed a significant difference (Table 2). The differences in the parasite burden in each group at two different times, a day after the end of medication administration, and the days before death were significant (Table 2).

**Table 2:** The parasite burden in different treatment groups by various doses of FLZ + SDZ/PYR

Groups	Days after challenge		*P* value
	One day after the end of medication	Last day (before death)	
Untreated	–	11,804,800	*P* < 0.001
SDZ/PYR 40/1	5,152,800	2,965,200	*P* < 0.001
*P* value	*P* < 0.009		
Untreated	–	11,804,800	*P* < 0.001
FLZ 10 + SDZ/PYR 40/1	1,324,400	1,052,000	*P* < 0.001
*P* value	*P* < 0.009		
Untreated	–	11,804,800	*P* < 0.001
FLZ 20 + SDZ/PYR 40/1	1,101,600	762,000	*P* < 0.001
*P* value	*P* < 0.009		

**Fig. 4:**
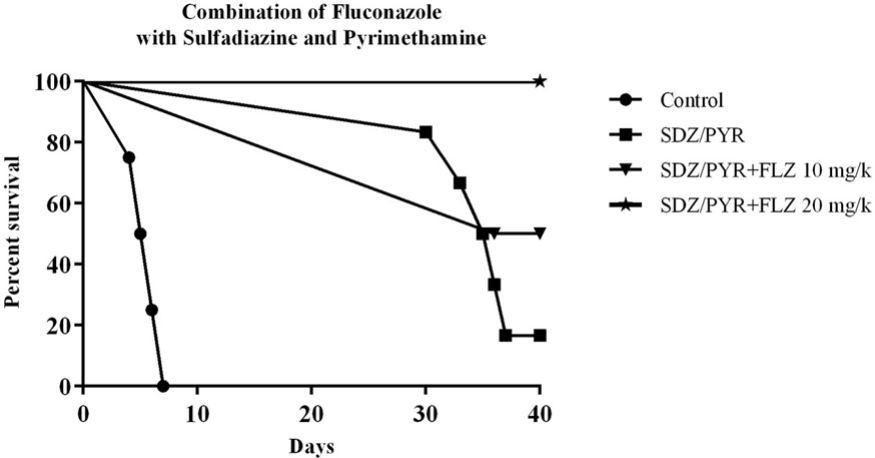
Effect of a 10-day treatment with 10 or 20 mg/kg/day of FLZ plus 40/1 mg/kg/day of SDZ/PYR on the survival rate of female BALB/c mice acutely infected with 10,000 tachyzoites of *T. gondii* RH strain. The results were evaluated by Kaplan–Meier product-limit method. *P* < 0.05 for the 10 or 20 mg/kg/day of FLZ plus 40/1 mg/kg/day of SDZ/PYR. Control; (*n* = 10); FLZ 10 (*n* = 10); FLZ 20 (*n* = 10)

The results of combining different doses of FLZ and SDZ/PYR showed a significant effect on the survival rate. In addition, the mortality prevention rate increased significantly in the mice treated in this group (Fig. 4). All mice treated with 20 mg/kg/day of FLZ in combination with 40/1 mg/kg/day of SDZ/PYR survived for 40 days. Indeed, combination therapy with FLZ plus 40/1 mg/kg/day of SDZ/PYR led to the highest survival rate (100%) and this value was significantly different as compared with the non-fluconazole (50%)-treated group (*P* = 0.0016). In addition, the mice treated with 10 mg/kg/day of FLZ plus 40/1 mg/kg/day of SDZ/PYR showed an increased survival rate in contrast to the non-fluconazole therapy, and at the end of 40 days, 90% of the mice in this group survived (*P* = 0.0032).

### Combination of FLZ and PYR

Due to the greater activity and power of PYR against the *Toxoplasma gondii* infection, different doses of FLZ with a fixed dose of PYR were examined. In this respect, the effects of the doses of 20, 40, and 80 mg/kg/day of FLZ and the dose of 10 mg/kg/day of PYR were examined (Table 3).

**Table 3:** The parasite burden in different group of treatment by various doses of FLZ + PYR

Groups	Day after challenge		*P* value
	Day after the end of medication	Last day (before death)	
Untreated	–	11,804,800	*P* < 0.001
PYR	1,870,800	916,000	*P* < 0.001
*P* value	*P* < 0.009		
Untreated	-	11,804,800	*P* < 0.001
FLU 20 mg + PYR	1,746,000	1,052,000	*P* < 0.001
*P* value	*P* < 0.009		
Untreated	–	11,804,800	*P* < 0.001
FLU 40 mg + PYR	1,592,400	1,556,000	*P* < 0.001
*P* value	*P* < 0.009		
Untreated	–	11,804,800	*P* < 0.001
FLU 80 mg + PYR	2,466,000	2,004,400	*P* < 0.001
*P* value	*P* < 0.009		

Results are the mean ± standard deviation of the quantity of DNA obtained from three different treatment groups. Kruskal–Wallis test compared the groups at the significance level of 0.05. Mann–Whitney *U* test was used for multiple comparisons between groups and different days. Bonferroni correction was used to change the significance level to 0.01 and 0.025. Parasite load of *Toxoplasma* tachyzoites indicated the statistically significant difference among *t* the two groups and different days

The lowest level of parasite loads was detected a day after the end of the treatment, 20 mg/kg/day of FLZ with 10 mg/kg/day of PYR, and the highest copy number was detected in the groups of 80 mg/kg/day of FLZ in combination with 10 mg/kg/day of PYR.

**Fig. 5:**
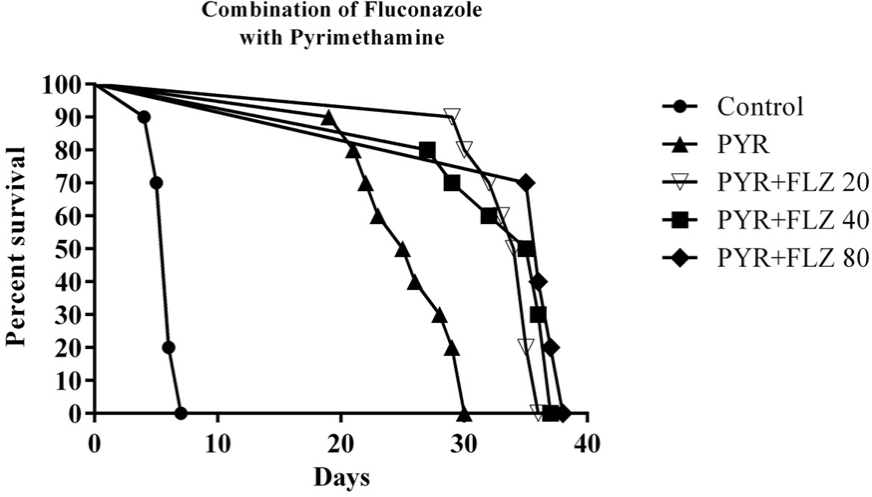
Effect of a 10 day treatment with 20, 40, and 80 mg/kg/day of FLZ plus 10 mg/kg/day of PYR on the survival of female BALB/c mice acutely infected with 10,000 tachyzoites of *T. gondii* RH strain. The results were evaluated by Kaplan–Meier product-limit method. *P* < 0.05 for the 20, 40, and 80 mg/kg/day of FLZ plus 10 mg/kg/day of PYR. Control; (*n* = 10); FLZ 20 (*n* = 10); FLZ 40 (*n* = 10); FLZ 80 (*n* = 10)

The constant dose of PYR 10 mg/kg/day in combination with different doses of FLZ increased the survival rate in the treated mice. The mortality prevention rate increased significantly in the mice treated with this group, but none of the mice in these treated groups survived until the end of the 40 days (Fig. 5). Median survival in the combination therapy of PYR and FLZ 20, 40, 80 10 mg/kg/ day was 34.5, 35.5, and 36 days (*P* < 0.05); in contrast, median survival in the treatment group was only 25.5 days with PYR (*P* < 0.05.

## Discussion

In the present study, after treating *Toxoplasma* infected mice, the parasite burden in the brain tissues was examined using the real-time qPCR method. All tissues collected during the study were positive in terms of infections. In addition, a survival study was performed on similar therapeutic groups. Data analysis showed the lowest level of the parasite burden and the highest death prevention rate in the mice group that received FLZ 20 mg/kg/day in combination with SDZ/PYR, at the doses of 40/1 mg/kg/day.

Research on market drugs is a convenient way of exploring more effective therapies against diseases and an alternative for previous treatments. FLZ is an antifungal agent that is well tolerated, and its side effects and liver toxicity are low, which is usually prescribed for the treatment of fungal infections in patients with HIV [17]. Due to its high solubility and fast and widespread distribution in body fluids, such as the cerebrospinal fluid, FLZ can reach the infection site in the central nervous system so easily and get activated against pathogens [17, 18]. The selective toxicity of Azole (Fluconazole, ketoconazole, and Itraconazole) against fungi is unique, because it disrupts the synthesis of ergosterol (a sterol fungal cell membrane) and does not damage cell membranes.

By inhibiting the production of ergosterol, Azole disrupts the permeability of the fungal cell membrane and ultimately destroys microorganisms [19–21]. In vitro, the effect of fluconazole and itraconazole on the reduction of IC50 was determined. In addition, the in vivo impact of fluconazole was approved to be anti-parasitic on the selective molecular target of *T. gondii* [19, 20]. The most effective method of treating TE is the use of known and confirmed combination therapies [22].

It has been established that combination therapy prevents the recurrence of the illness in humans, in contrast to monotherapy with PYR [23]. In addition, using combination therapy, the dose of anesthetic drugs decreases, but the efficacy of these drugs, drug consumption, side effects, and toxicity do not decrease [24]. Therefore, finding new combination therapies is required for the treatment of toxoplasmosis. However, in this experiment, the combination doses of SDZ/PYR were used, as prescribed in the TE clinical treatment. The doses included 50–100 mg/day of PYR and 4–8 g/day of SDZ [15, 25]. Nevertheless, the doses ranged from 40 to 160/1 of SDZ/PYR; nevertheless, the dose we chose was 40/1 mg/kg/day [15].

Formerly, Giemsa staining and light microscopic examinations were selected to assess the parasitic load, the severity of the infection, and the impact of anti-parasitic drugs and vaccines. However, the staining method for the detection of the parasite burden is inefficient, because it is time-consuming and suffers insignificant sensitivity and ocular errors compared with molecular techniques, especially the real-time QPCR [26]. The QPCR method is highly sensitive and can determine the exact amount of the parasitic load in the blood and various tissues, even at the lowest amount of 0.05 parasites per reaction [12, 27].

Data have so far been published on the parasite burden determined in various tissues after medication administration or on the effect of anti-parasitic vaccines by the real-time QPCR method [28, 29].

In the present study, the real-time QPCR method was used, and the targets of the B1 gene and the parasitic load in the brain of the infected mice were identified; in addition, in the same treatment groups, survival changes were evaluated after medication administration. The results suggested that the combined groups, including FLZ plus SDZ/PYR, and PYR combined with FLZ were effective against *T. gondii* in vivo and reduced the parasite burden significantly. In a study by Martins-Duarte et al., the anti-parasitic effects of FLZ in the mice infected with the ME49 strain and the lack of effects on RH strains were reported. The severity of the disease in the mice infected with the RH strain has been a concern [19].

In addition, it was approved that specific treatment regimen including FLZ in combination with SDZ/PYR or PYR with FLZ led to the up-regulation of survival rate, and this treatment prevented the relapse and death caused by the RH strain, compared with SDZ in combination with PYR or PYR without FLZ [15].

The results of the combination of the doses 10 and 20 mg/kg/day of FLZ with 40/1 of SDZ/PYR are similar to the treatment of 20 mg/kg/day of FLZ in combination with 10 mg/kg/day of PYR, thereby indicating a reduction in the parasite burden compared to the combination therapy of SDZ/PYR without FLZ. The results are interesting due to the reduction in the dosage of SDZ as well as the side effects. However, the increased parasite burden in the treatment doses of 40 and 80 mg/kg/day of FLZ with PYR compared to the dose of 20 mg/kg/day of FLZ with PYR showed that the high doses of FLZ with PYR had antagonistic activities. The survival results of this study showed similarities/differences with the study conducted in 2013 by [15], where the combination of FLZ, PYR, and SDZ was significantly different from the non-FLZ therapy. The mice treated with SDZ and PYR combined with 20 mg/kg/day of FLZ up until the end of the 40 days had the highest protection against death and recurrence of the disease. The results of the survival study in FLZ treatment groups along with PYR showed a significant difference in survival and death prevention, but contrary to the study by Martins, the mice did not survive until the end of the 40-day review. The significant effects of reducing the parasitic burden and increasing the survival rate in combination therapy groups indicate the dependence of these results on the dose of fluconazole, in contrast to non-fluconazole treatments. However, the parasite burden decreased by the FLZ combination therapy with SDZ/PYR, thereby increasing the dose of FLZ; in addition, it was demonstrated that the effects depended on the dose of FLZ. Besides, the combinations of FLZ with SDZ/PYR and PYR showed a synergistic effect, but when the combination therapies were evaluated, they exerted an increasing effect only in vitro [15].

A survey conducted on the combination of clindamycin with PYR did not show positive results in terms of synergistic effects in vitro [30], but the compound was effective in the body in the treatment of TE patients, because drug interactions could not be ignored, as FLZ is known as an inhibitor of cytochrome P450 oxidative metabolism. FLZ is a potent non-competitive inhibitor for the isoform 2C9 of cytochrome and can interfere in the biotransformation of other drugs [31]. A study conducted by Winter et al. in vitro, using human liver microcosms, showed that CYT2C9 was effective in metabolizing SDZ in terms of its hydroxylamine intermediates [32].

Hence, an increase in the combined effect of SDZ/PYR plus FLZ could have been due to the increase in SDZ in the blood concentration. The activity of FLZ in the body decreases SDZ metabolism. There is little information about PYR metabolism, though it is believed that it occurs in the liver. PYR metabolism is done by the CYP oxidative enzyme, so exploring the reactions in vitro is not possible [33].

In the end, the combination of FLZ and SDZ/PYR could be a remedy for the speedy recovery and prevention of toxoplasmosis relapse. Of course, this conclusion has no proof as a follow-up of the infected mice for only 40 days is not enough period to reach that conclusion and further investigations are needed to get more results regarding another toxoplasmosis treatment aspect.

Moreover, in patients intolerant to SDZ, PYR could be used in combination with FLZ as an alternative treatment. In addition, experiments on other animal models are required to approve this combination treatment for the case of toxoplasmosis in humans.

Collectively, our findings demonstrate that FLZ combination therapy has the potential to be a good candidate for acute toxoplasmosis treatment. Before clinical applications, further studies are required in vivo to explore and optimize the toxicity, efficacy, and minimizing their side effect. Finally, future perspectives to be considered include work on different strains of *Toxoplasma* especially cystogenic strains to obtain comprehensive data regarding these agents’ anti *toxoplasma* activity.

## Data Availability

That if the paper contains material (data or information in any other form) that is the intellectual property and copyright of any person(s) other than the Author(s), then permission of the copyright owner(s) to publish that material has been obtained, and is clearly identified and acknowledged in the text of the paper.
